# Long-Term Risk of Pancreatic Cancer After Acute Acetylcholinesterase Inhibitor Insecticide Exposure: A Nationwide Cohort Study

**DOI:** 10.3390/curroncol32100528

**Published:** 2025-09-23

**Authors:** JeongMi Moon, EuJene Jung, ByeongJo Chun, DongKi Kim, YeonJi Seong

**Affiliations:** 1 Department of Emergency Medicine, Chonnam National University Hospital, Gwangju 61487, Republic of Korea; 2 Department of Emergency Medicine, Chonnam National University Medical School, Hak Dong 8, Gwangju 61469, Republic of Korea; 3 Department of Emergency Medicine, Chonnam National University Hwasun Hospital, Hwasun 58128, Republic of Korea

**Keywords:** acetylcholinesterase, carbamate, organophosphate, pancreatic cancer

## Abstract

This nationwide cohort study investigated whether a single episode of acute exposure to high-dose acetylcholinesterase (AChE) inhibitor insecticide is associated with an increased risk of pancreatic cancer. Data from the Korean National Health Insurance Service were analyzed. The case group (*n* = 938) included adults exposed to organophosphate or carbamate insecticide and the control group *(n* = 3752) was matched by age, sex, and socioeconomic status. Cox proportional hazards regression was used to calculate hazard ratios (HRs) and 95% confidence intervals. Kaplan–Meier curves with log-rank tests evaluated differences in pancreatic cancer incidence. Over 33,219.8 person-years of follow-up, pancreatic cancer developed in 9 patients in the case group and 19 patients in the control group. The cumulative incidence of pancreatic cancer was significantly higher in the case group (log-rank *p* < 0.01). Acute high-dose exposure to AChE inhibitor insecticide was associated with an increased risk of pancreatic cancer (adjusted HR: 2.57). The risk was particularly elevated among women (HR: 5.85) and individuals with diabetes (HR: 2.75). Acute high-dose exposure to AChE inhibitor insecticide may increase the risk of pancreatic cancer. Women and those with diabetes may represent high-risk subgroups. These findings highlight the need for targeted cancer surveillance and further confirmatory studies.

## Simple Summary

Although pesticide exposure has long been known to harm human health, it has not been clear whether a single episode of severe exposure to acetylcholinesterase inhibitor insecticide could have lasting effects. By analyzing nationwide Korean health records, this large study found that people who experienced one acute high-dose exposure to acetylcholinesterase inhibitor insecticide had a significantly higher chance of later developing pancreatic cancer. The risk was especially high among women and those with diabetes. These results suggest that such exposure may act as a hidden long-term pancreatic cancer risk. The findings highlight the importance of monitoring exposed individuals and guiding public health authorities to strengthen pesticide safety regulations. They further encourage physicians to consider implementing pancreatic cancer surveillance in high-risk patients and inspire future researchers to explore how acute toxic events may trigger pancreatic cancer.

## 1. Introduction

Globally, and particularly in South Korea since 2002, the incidence and mortality rates of pancreatic cancer have steadily increased [1,2]. In South Korea, pancreatic cancer constitutes the eighth-most common cancer, with an age-standardized incidence of 17.8 per 100,000. Importantly, it had the lowest 5-year relative survival rate (16.5%) between 2018 and 2022 [3]. Because diagnosis frequently occurs at an advanced stage, surgical resection is often not feasible. Consequently, delayed detection remains a major barrier to improved outcomes, underscoring the urgent need to identify risk factors that may facilitate screening and early detection [4]. Surveillance of high-risk populations has shown potential for earlier diagnosis and improved clinical outcomes [5].

Carbamate and organophosphate (OP) insecticides inhibit the acetylcholinesterase (AChE) enzyme and are widely used in agriculture and indoor pest control. Over the past decade, several case–control and cohort studies have shown that occupational exposure to these pesticides is associated with increased risks of cancers such as breast cancer, lung cancer, melanoma, and non-Hodgkin lymphoma [6]. Specifically, growing evidence implicates their potential contribution to the development of pancreatic cancer. Chronic exposure to dichlorvos or parathion exhibits carcinogenic effects on the pancreas in rat models [7,8]. Long-term carbamate exposure substantially reduces the area of Langerhans islets in rats [9]. Furthermore, urinary concentrations of OP compounds are considerably higher in patients with pancreatic ductal adenocarcinoma than in healthy controls [10].

However, the extrapolation of findings from chronic low-dose occupational pesticide exposure to cases of acute pesticide exposure in South Korea is particularly challenging. In South Korea, approximately 85% of pesticide poisoning cases result from intentional ingestion of high doses for suicidal purposes [11]. Pesticide-induced toxicity depends on both the duration and concentration of exposure [12], and acute high-dose exposures may elicit distinct biological consequences compared with chronic occupational exposure.

Acute high-dose OP exposure has been shown to cause rapid and severe cellular damage, as well as trigger inflammatory responses in the pancreas [13]. Experimental studies also demonstrated that acute high-dose malathion exposure, at concentrations far exceeding environmental levels, significantly reduces viability of rodent pancreatic cells; acute low-dose exposure does not induce cell death [12]. These findings are consistent with clinical observations of pancreatic injury—ranging from mild to necrotizing pancreatitis following acute intentional exposure to OP or carbamate insecticides [14].

Despite the biological plausibility and accumulating evidence linking chronic pesticide exposure to cancer, no population-based cohort study has assessed the long-term risk of pancreatic cancer after acute exposure to high-dose AChE inhibitor insecticide. Clarifying this association may provide critical insights into the etiology of pancreatic cancer and help identify high-risk populations. It may also inform public health interventions, including targeted surveillance programs. Given the poor prognosis of pancreatic cancer and the unique exposure profile in South Korea, this study aimed to assess whether acute AChE inhibitor insecticide exposure increases the subsequent risk of pancreatic cancer and to identify potentially high-risk subgroups.

## 2. Methods

### 2.1. Data Source and Ethics

We conducted a population-based retrospective cohort study using the National Health Insurance Service (NHIS) database, which operates within Korea’s single-payer healthcare system. Over 97% of Korean residents are enrolled in the NHIS, and approximately 3% of individuals in the lowest income bracket receive Medical Aid, which is incorporated into the NHIS. This comprehensive structure enables near-complete capture of patient demographics (e.g., age, sex), diagnostic codes based on the International Statistical Classification of Diseases and Related Health Problems, Tenth Revision (ICD-10), medical procedures, and prescription records. The database therefore provides a robust resource for population-based epidemiological and clinical investigations.

In this study, anonymized patient identification numbers were used to track healthcare utilization, including outpatient visits, hospital admissions, procedures, and prescription medications. All personally identifiable information in the database is encrypted, ensuring that researchers had no access to identifiable data. Because the data were deidentified and retrospectively analyzed, individual informed consent was not required. The study was approved by the Institutional Review Board of our institution (IRB No.: CNUH-EXP-2024-073), and a waiver of informed consent for the use of secondary data was granted in accordance with local ethical guidelines.

### 2.2. Study Population

The study cohort was extracted from the NHIS database, covering the period from 1 January 2014, to 31 December 2022. Between 2014 and 2015, adult patients aged 18 years or older without prior diagnoses of the toxic effects of OP and carbamate insecticides (ICD-10 code T60.0), acute or chronic pancreatitis, or pancreatic cancer were considered for inclusion. Individuals without a T60 code during the entire observation period were assigned to the control group, and each was given a randomly assigned index date matched to the distribution of case group diagnosis dates. Controls were matched to cases at a 4:1 ratio based on age, sex, and socioeconomic status. Follow-up began on the index date and continued until death, emigration, or the end of the observation period. Individuals diagnosed with pancreatic cancer between 2014 and 2015 were excluded under the assumption that undiagnosed cases may not have sought medical care.

In South Korea, the Ministry of Employment and Labor oversees the registration system related to health effects associated with occupational pesticide exposure. Within the health insurance system, a diagnosis coded as T60–69.0 represents an episode of acute exposure to pesticide [15].

### 2.3. Primary Outcome and Definitions

The primary outcome of this study was newly diagnosed pancreatic cancer (i.e., diagnosis of new-onset pancreatic cancer). Pancreatic cancer was identified using ICD-10 code C25.9, which corresponds to malignant neoplasm of the pancreas, unspecified. A diagnosis was considered confirmed if the patient was hospitalized under this code or if at least two outpatient visits were recorded under the same code. In Korea, the NHIS administers the Rare and Intractable Disease (RID) registration program. Patients with oncological diseases are eligible for a 90% copayment reduction under this program. To qualify, all patients must undergo evaluation by a board-certified specialist according to uniform, government-mandated diagnostic criteria.

### 2.4. Data Collection

Demographic variables (index date and sex), socioeconomic status, and comorbidity profiles were obtained from the NHIS database. Socioeconomic status was determined on the basis of income level, stratified into quartiles using health insurance premium data. Comorbidities, including diabetes mellitus (ICD-10: E10–E14), hypertension (I10), dyslipidemia (E78), and chronic kidney disease (N18) were included as confounding variables because of their established associations with pancreatic cancer incidence [16]. Mental disorders were defined according to ICD-10 codes F01–F99

### 2.5. Statistical Analysis

After individuals had been matched by age, sex, and insurance level at a 1:4 ratio, baseline demographic characteristics were compared between the case and control groups. Categorical variables were summarized as frequencies and percentages, then compared using chi-square tests. Continuous variables were summarized as means with standard deviations or as medians with interquartile ranges, then compared using either Student’s t-test or the Wilcoxon rank-sum test, as appropriate.

The crude 8-year incidence rate (2014–2022) of pancreatic cancer was calculated per 1000 person-years according to AChE inhibitor insecticide exposure status. Hazard ratios (HRs) and 95% confidence intervals (CIs) were estimated using Cox proportional hazards regression models with fixed covariates to assess relative risk over the 8-year period. Cumulative incidence was analyzed with Kaplan–Meier curves, and differences between groups were evaluated with the log-rank test. Multicollinearity among covariates was assessed to confirm model validity.

All statistical analyses were performed using SAS version 9.4 (SAS Institute Inc., Cary, NC, USA). Two-sided *p*-values < 0.05 were considered statistically significant.

## 3. Results

### 3.1. Clinical Characteristics of the Study Population

The baseline characteristics of the 938 case patients and 3752 matched controls are summarized in Table 1. The two groups were well balanced with respect to age, sex, socioeconomic status, and major comorbidities, including hypertension, diabetes, coronary artery disease, and chronic kidney disease. Mental disorders, however, were significantly more prevalent in the case group (20.0%) compared with the control group (13.5%, *p* < 0.01).

### 3.2. Main Outcomes

**Figure 1:**
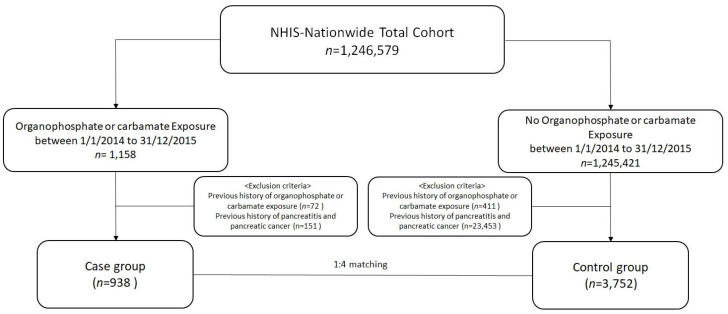
Flowchart of study design. NHIS, National Health Insurance Service.

**Figure 2:**
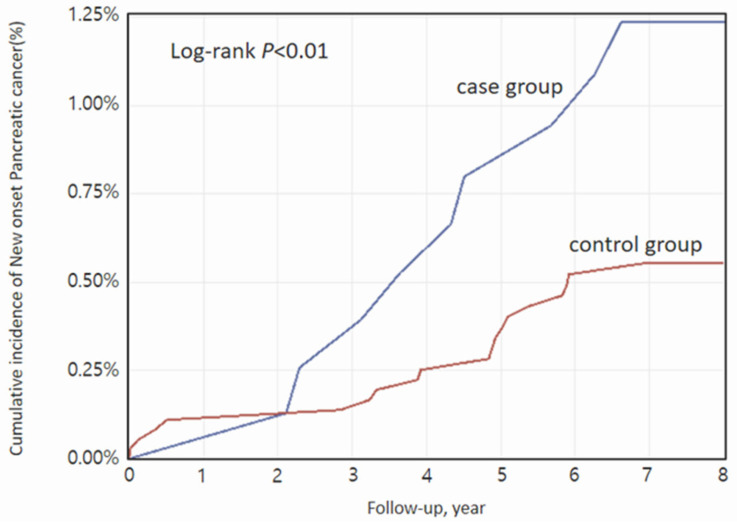
Kaplan–Meier plot of the cumulative incidence of pancreatic cancer associated with acute exposure to AChE inhibitor insecticide.

During a total follow-up of 33,219.8 person-years (median, 6.7 years; IQR, 4.5–7.9; maximum, 8 years after a 1-year lag), 28 cases of pancreatic cancer were diagnosed—9 in the case group and 19 in the control group (Figure 1, Table 2). Kaplan–Meier survival curves showed a significantly higher cumulative incidence of pancreatic cancer in the case group (log-rank *p* < 0.01) (Figure 2). In multivariable conditional logistic regression analysis, which adjusted for potential confounders including hypertension, diabetes mellitus, coronary artery disease, chronic kidney disease, and mental disorders, acute exposure to AChE inhibitor insecticide was associated with more than a twofold increased risk of pancreatic cancer compared with the control group (adjusted HR, 2.57; 95% CI, 1.15–5.75).

### 3.3. Subgroup Analysis

Stratified analyses were performed according to age, sex, hypertension, and diabetes status (Table 3). The associations between acute exposure to AChE inhibitor insecticide and pancreatic cancer were not statistically significant across age groups (*p* = 0.23) or among individuals with or without hypertension (*p* = 0.07). However, the risk was significantly elevated among women (adjusted HR, 5.85; 95% CI, 3.49–13.01). Participants with diabetes also showed a significantly increased risk (HR, 2.75; 95% CI, 1.14–6.63).

## 4. Discussion

In this large nationwide cohort study, acute exposure to AChE inhibitor insecticide was significantly associated with an increased risk of pancreatic cancer, with particularly elevated risks among women and individuals with diabetes mellitus. To our knowledge, this is the first population-based study to demonstrate this association.

The observed adjusted HR of 2.57 for acute exposure to AChE inhibitor insecticide indicates a substantial association with pancreatic cancer, underscoring its potential clinical and public health significance as a critical risk factor. In contrast to the inconsistent findings reported for chronic occupational exposure, evidence on acute high-dose exposure to AChE inhibitor insecticides remains limited, highlighting the novelty and relevance of our results.

Although this study demonstrated an association, it did not establish causality between acute exposure to AChE inhibitor insecticide and pancreatic cancer. Nevertheless, the link is biologically plausible. Chemicals can initiate, promote, and drive carcinogenesis by inducing DNA damage and oxidative stress and by suppressing immunosurveillance [17]. Acute exposure to OP induces oxidative stress and chromosomal DNA damage independent of oxidative stress, both of which persist after treatment of acute exposure [18,19,20,21,22,23]. In mice, acute exposure to a low dose of an OP degradation metabolite causes immunotoxicity that lasts up to 20 days after exposure. This immunomodulatory effect impairs immune responses against tumor cells, thereby promoting tumor growth [24]. Similarly, carbamates alter gene expression, induce structural and functional changes in immune cell populations, and downregulate the antioxidant defense system [25,26]. Histopathological changes in the pancreas after two months of dimethoate exposure have been reported to be irreversible [22]. Acute irreversible pancreatic injury, such as fatty pancreas or direct acinar damage, may predispose individuals to malignancy. These carcinogenic effects of acute exposure to AChE inhibitor insecticide, which may persist after hospital discharge, could synergistically contribute to the onset of pancreatic cancer across a patient’s lifetime. Although these mechanistic findings strengthen the plausibility of a causal link, our study design cannot confirm causation. Future investigations employing prospective cohort designs, biomarker-based exposure assessments, and experimental models will be essential to assess whether acute AChE inhibitor insecticide exposure directly contributes to pancreatic carcinogenesis. Such studies are critical for advancing from observed associations toward establishing causality

In the present study, among individuals with diabetes, acute exposure to AChE inhibitor insecticide was associated with a twofold increased risk of pancreatic cancer. Previous case reports and animal studies have shown that exposure to OPs or carbamates induces transient insulin resistance and disrupts glucose homeostasis [9,27]. Notably, acute glucose dysregulation may persist for 3–12 months after hospital discharge after acute exposure to OP [28]. Hyperglycemia and insulin resistance are recognized promoters of pancreatic carcinogenesis through mechanisms such as providing excess energy to cancer cells, stimulating epidermal growth factors, and activating insulin-like growth factor 1 (IGF-1) receptor-mediated pathways [29,30,31]. Acute AChE inhibitor insecticide exposure may therefore exacerbate diabetes-related metabolic disturbances, fostering a microenvironment conducive to pancreatic cancer development.

Although pancreatic cancer incidence is generally higher in men than in women [1], this study revealed a fivefold increase in risk among women after acute exposure to AChE inhibitor insecticide. This elevated risk may reflect the estrogen-disrupting properties of these pesticides, which interfere with key cellular processes, including proliferation, metastasis, and apoptosis, thereby facilitating cancer progression [32,33,34]. In vivo studies have shown that estrogen treatment can substantially suppress the progression of precancerous pancreatic lesions [33].

Given that the majority of acute pesticide exposures in South Korea result from intentional ingestion for suicide attempts, the higher prevalence of mental disorders in the case group is not unexpected. This observation aligns with previous domestic studies examining patients with acute pesticide exposure [35]. Several investigations, including a large-scale prospective study, have reported no causal association between mental disorders and the incidence of pancreatic cancer [36,37]. In the present study, acute exposure to AChE inhibitor insecticide remained significantly associated with pancreatic cancer risk even after adjusting for mental disorders as covariates in multivariable analyses. Nevertheless, mental disorders may contribute to delays in cancer diagnosis and poorer treatment compliance, leading to higher mortality among patients with cancer [38]. Considering the high prevalence of mental disorders among individuals with acute exposure to AChE inhibitor insecticides, these findings underscore the importance of pancreatic cancer surveillance in this particularly vulnerable population.

In contrast to our findings, several previous epidemiological studies based on self-reported questionnaires reported no association between occupational exposure to AChE inhibitor insecticides and pancreatic cancer [39,40,41]. These discrepancies may reflect differences in the type of insecticide exposure (chronic low-dose vs. acute high-dose), ethnicity, or modifiable risk factors such as diet and obesity.

This study had several limitations. First, residual confounding may still be present. We lacked information on genetic susceptibility and lifestyle behaviors, including smoking, alcohol consumption, obesity, and dietary patterns, each of which is a well-established determinant of pancreatic cancer. Socioeconomic status was matched between the case and control groups, which partially addresses lifestyle factors in Korea [31]. Nevertheless, despite this matching, the absence of direct adjustment for these risk factors remains an important limitation. Future studies should directly assess these pancreatic cancer risk factors to confirm and further develop these findings. Second, the generalizability of the results may be limited. Pesticide formulations, patterns of use, and regulatory frameworks differ substantially across countries. Moreover, given that the South Korean government has implemented restrictions on highly toxic pesticides, the OP or carbamate agents implicated in this study may not represent those currently in use. Multinational studies in diverse populations with varying ethnic backgrounds, lifestyle characteristics, and pesticide regulations are warranted to determine the global applicability of these findings. Third, from a public health perspective, our findings indicate that individuals with a history of acute AChE inhibitor insecticide exposure—particularly women and those with pre-existing diabetes—may constitute a high-risk group for pancreatic cancer and could benefit from targeted screening and structured follow-up after hospital discharge. However, our study only demonstrated an association and did not capture the timing of cancer onset following exposure. As effective surveillance requires evidence on the optimal timing for screening, further longitudinal studies are warranted to determine the appropriate timing and methodology for implementing such strategies should be implemented to improve early detection in these high-risk groups. Fourth, the NHIS database provides diagnostic information based on ICD-10 codes, in which poisoning caused by OP and carbamate insecticides is grouped under a single code (T60.0). Information regarding the specific causative substance is not included. Consequently, we were unable to identify the exact pesticide involved. However, a previous study based on data from 20 hospitals in South Korea indicated that the ratio of OP to carbamate exposure in adults from 2013 to 2014 was 4:1 [42]. The leading causes of acute exposure to OP insecticides were fenithrothion (22.4%), dichlorvos (20.9%), and diazinon (13.4%), whereas methomyl represented 54.5% of acute exposure to carbamate in 2014 [42]. Fifth, the statistical power of the study was limited. Only 28 cases of pancreatic cancer were observed—9 in the case group and 19 in the control group. Consequently, the CIs around the HRs were wide, indicating potential instability in the estimates. Nevertheless, the incidence of acute pesticide exposure in South Korea is approximately 15.4 per 100,000 population, while the age-standardized incidence of pancreatic cancer is 17.8 per 100,000 [3,43]. Pesticides comprise a range of chemical agents, including insecticides, herbicides, fungicides, and related compounds. Given that acute exposure to AChE inhibitor insecticide was the inclusion criterion and pancreatic cancer was the primary outcome, the relatively small number of pancreatic cancer cases observed in our cohort may reflect the inherent epidemiologic characteristics. To address this limitation, studies with larger cohorts and integrated collaborative databases are warranted to confirm these findings and to investigate differential risks by sex, metabolic factors, and other potential modifiers.

In conclusion, acute exposure to high-dose AChE inhibitor insecticide was associated with an increased risk of pancreatic cancer, with particularly high relative risks observed among women and individuals with diabetes mellitus These findings, although requiring confirmation in future studies, highlight the need for the development and implementation of structured pancreatic surveillance programs for these high-risk patients, especially those who are women or have diabetes, given the poor prognosis of pancreatic cancer due to late detection.

## Figures and Tables

**Table 1:** Characteristics of the control and case groups in this study.

Variables	All	Case Group	Control Group	*p*-Value
	N (%)	N (%)	N (%)	
All	4690 (100.0)	938 (100.0)	3752 (100.0)	
Age (years)				1.00
18–40	300 (6.4)	60 (6.4)	240 (6.4)	
41–60	2020 (43.1)	404 (43.1)	1616 (43.1)	
61–80	2000 (42.6)	400 (42.6)	1600 (42.6)	
80–120	370 (7.9)	74 (7.9)	296 (7.9)	
Sex				1.00
Male	3190 (68.0)	638 (68.0)	2552 (68.0)	
Female	1500 (32.0)	300 (32.0)	1200 (32.0)	
Socioeconomic status				1.00
Q1 (Lowest)	940 (20.0)	188 (20.0)	752 (20.0)	
Q2	710 (15.1)	142 (15.1)	568 (15.1)	
Q3	845 (18.0)	169 (18.0)	676 (18.0)	
Q4	1135 (24.2)	227 (24.2)	908 (24.2)	
Q5 (Highest)	1060 (22.6)	212 (22.6)	848 (22.6)	
Comorbidities				
Hypertension, yes	1478 (31.5)	262 (27.9)	1216 (32.4)	0.01
Diabetes, yes	797 (17.0)	139 (14.8)	658 (17.5)	0.05
CAD, yes	339 (7.2)	57 (6.1)	282 (7.5)	0.13
CKD, yes	209 (4.5)	29 (3.1)	180 (4.8)	0.02
Mental disorder, yes	694 (14.8)	188 (20.0)	506 (13.5)	<0.01

Q, quintile; CAD, coronary artery disease; CKD, chronic kidney disease.

**Table 2:** Incidence rate of pancreatic cancer and multivariable conditional logistic regression analysis of pancreatic cancer.

	Number at Risk	Cancer Events	Person-Years	Incidence Rate per 1000 PYs	Model 1	Model 2	Model 3
					cHR (95% CI)	aHR (95% CI)	aHR (95% CI)
Exposure							
No	3752	19	27,331.5	0.70	ref.	ref.	ref.
Yes	938	9	5888.3	1.53	2.41 (1.09–5.34)	2.61 (1.17–5.81)	2.57 (1.15–5.75)

PY, person-year; cHR, crude hazard ratio; aHR, adjusted hazard ratio; CI, confidence interval; ref., reference. Model 2: adjusted hypertension and diabetes. Model 3: Model 2 + coronary artery disease, chronic kidney disease, and mental disorder.

**Table 3:** Hazard ratios of pancreatic cancer associated with acute exposure to AChE inhibitor insecticide in subgroup analysis.

	Number at Risk	Cancer Events	HR (95% CI)	*p*-Value
Age group (years)				0.23
18–60	464	6	4.03 (0.65–25.1)	
61–120	474	3	2.39 (0.97–5.89)	
Sex				<0.01
male	638	4	0.99 (0.29–3.41)	
female	300	5	5.85 (3.49–13.01)	
Hypertension				0.07
No	676	6	2.02 (0.65–6.27)	
Yes	262	3	3.48 (1.03–11.68)	
Diabetes				<0.01
No	799	7	1.52 (0.17–13.22)	
Yes	139	2	2.75 (1.14–6.63)	

## Data Availability

The data supporting the findings of this study were obtained from the National Health Insurance Service of Korea. Access restrictions apply, as these data were used under license for the present study and are not publicly available. The corresponding author will make data available upon reasonable request and with permission from the National Health Insurance Service of Korea.
